# Copyrolysis of Biomass and Coal: A Review of Effects of Copyrolysis Parameters, Product Properties, and Synergistic Mechanisms

**DOI:** 10.1155/2016/6197867

**Published:** 2016-09-18

**Authors:** Cui Quan, Ningbo Gao

**Affiliations:** Department of Environmental Science and Engineering, School of Energy and Power Engineering, Xi'an Jiaotong University, Xi'an, Shaanxi 710049, China

## Abstract

Concerns in the last few decades regarding the environmental and socioeconomic impacts of the dependence on fossil fuels have resulted in calls for more renewable and alternative energy sources. This has led to recent interest in copyrolysis of biomass and coal. Numerous reviews have been found related to individual pyrolysis of coal and biomass. This review deals mainly with the copyrolysis of coal and biomass and then compares their results with those obtained using coal and biomass pyrolysis in detail. It is controversial whether there are synergistic or additive behaviours when coal and biomass are blended during copyrolysis. In this review, the effects of reaction parameters such as feedstock types, blending ratio, heating rate, temperature, and reactor types on the occurrence of synergy are discussed. Also, the main properties of the copyrolytic products are pointed out. Some possible synergistic mechanisms are also suggested. Additionally, several outlooks based on studies in the literature are also presented in this paper.

## 1. Introduction

Coal is the most abundant fossil fuel energy source available to the world economy and its reserve was expected to last for up to 200 years compared to about 65 years and 40 years for natural gas and crude oil, respectively. The pyrolysis of coal is a good method for producing liquid fuels and other chemicals; however, the yields of these products are limited due to low content of hydrogen in coal. Hydropyrolysis, a pyrolysis process under hydrogen, is an effective method to improve tar yield and quality, but high cost of pure hydrogen hinders its industrial application. Accordingly, it is necessary to supply H_2_ for coal from other hydrogen-rich materials, such as plastic wastes, polymers, petroleum residues, and coke-oven gas.

**Figure 1:**
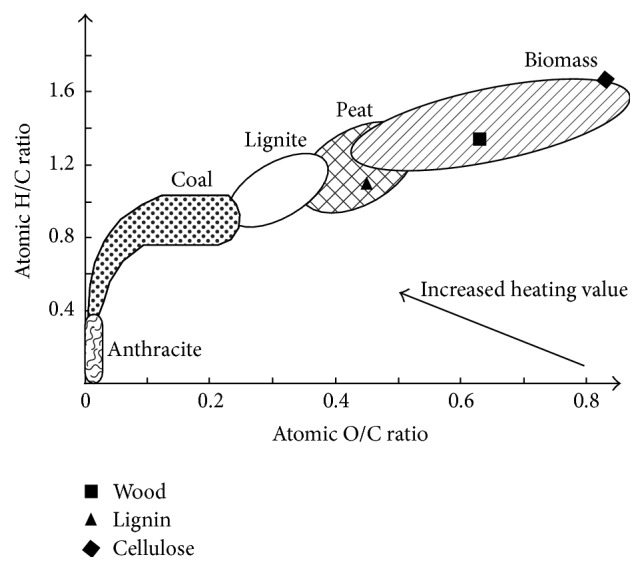
Van Krevelen's diagram showing the various H/C ratios and O/C ratios for different feedstocks [1].

Compared with plastic wastes and so forth biomass is a perspective source to replace fossil fuels in the future, as it is abundant, renewable, clean, and carbon dioxide neutral. Both biomass and coal are carriers of accumulated solar energy. The formation profile, however, changes the nature and availability of the two fuels. The difference in composition from biomass to coal is illustrated using a Van Krevelen diagram in terms of hydrogen/carbon (H/C) and oxygen/carbon (O/H) ratios as described in Figure 1 [1]. It could be clearly observed that, in comparison to coal, biomass has a higher value of H/C ratio (1.26–1.58) and O/C ratio (0.4–0.8). Higher hydrogen contents in biomass indicated that biomass could act as hydrogen donors in copyrolysis with coal. In addition, pyrolysis is inherently meant to be completed in an inert atmosphere, while the presence of higher oxygen content in biomass actually provides a significant increase in reactivity of the pyrolysis environment, facilitating the conversion of coal. Thus, some synergy effect may be expected when coprocessing biomass with coal.

Recently, many researchers used different fuels (such as sawdust, legume straw, lignite, and bituminous coal) and different reactors (such as TGA, fluidized bed reactor, fixed-bed reactor, and free fall reactor) under various operating parameters (such as temperature, heating rate, blending ratio, particle size, and contacting way of particles) to study the copyrolysis behaviors focusing on product distribution and product characteristics, as well as the possible existing synergetic effects. In general, to evaluate the interaction between biomass and coal blend, the experimental values were compared with the theoretical values which are sum of the values of individual samples in proportion to their blending values. The percentage of increasing or decreasing of the experimental values with respect to the theoretical values is called synergetic effects. The lack of synergy between the two fuels during copyrolysis is indicated by the linear relationship between the volatile matter release/product yield and the percentage of biomass added to the mixture. Given such a wide range of variables involved, the results obtained by different groups are sometimes conflicting. That is to say, synergistic or additive behaviors were reported to occur for copyrolysis processes of biomass/coal blends. In a study by Vuthaluru [2], the pyrolysis of biomass and coal blends by thermogravimetric analysis (TG) in different biomass/coal ratios showed no synergy effect, and there is a linear relationship between the char yield and the amount of biomass in the blend. Pan et al. [3] and Kastanaki et al. [4] also confirmed that no interaction took place between biomass and coal in a blend during pyrolysis. More recent efforts by Aboyade et al. [5], Chen et al. [6], Shui et al. [7], Park et al. [8], Ulloa et al. [9], and Haykiri-Acma and Yaman[10] have challenged this view, and they show that there are significant interactions between the coal and biomass fractions during pyrolysis in TG. Onay et al. [11], Sonobe et al. [12], Zhang et al. [13], Wei et al. [14, 15], Yuan et al. [16], and Li et al. [17] also verified the occurrence of synergy effect on the yields of the major pyrolytic products, gaseous component, tar components, and the reactivities of the chars. Interestingly, Park et al. [8] observed that synergy occurs on both TG and fixed-bed reactor, while Sonobe et al. [12], who conducted the copyrolysis of Thai lignite and corncob, verified the presence of synergy effect on a fixed-bed reactor rather than a TG apparatus. In addition, researchers who study the distribution of major products such as char, liquid, and gas tend to find no evidence of synergy effect [18], while those who study the composition of the volatiles tend to conclude the opposite [12, 19]. The occurrence of synergy during copyrolysis is generally not conclusive, and it depends on the pyrolysis technique and fuels used. These conflicting conclusions are intriguing and need to be clarified. Numerous reviews have been found related to individual studies on coal pyrolysis and biomass pyrolysis [20–22]. To our knowledge, little reviews are available on copyrolysis of coal/biomass blends [23, 24].

In this paper, a comprehensive overview on copyrolysis of coal and biomass was presented. The interest is to focus on the synergistic effects or the additive effect between coal and biomass in their copyrolysis. The effects of reaction parameters such as feedstock types, blending ratio, heating rate, temperature, and reactor types on the occurrence of synergy and on the distribution and properties of copyrolytic products are pointed out. Moreover, some possible synergistic mechanisms during copyrolysis of coal/biomass blends were also presented.

## 2. Copyrolysis Reaction Parameters

Copyrolysis of biomass and coal blend generally goes through a series of extremely complex reactions. Many pyrolysis process parameters such as feedstock types, blending ratio, heating rate, temperature, and reactor types may strongly affect the yield and properties of the products.

### 2.1. Effect of Feedstock Types

The types of blending fuels ought to be a major factor that can intrigue the synergy. It has been shown that many blends of biomass species and coal, such as hazelnut shell and coal [25], legume straw and coal [13], sawdust and coal [26], microalgae and coal [6], corncob and coal [12], and corn stalk and subbituminous coal [27], exhibit synergetic effects during copyrolysis process.

Biomass is mainly composed of several chemical constituents: cellulose, hemicellulose, lignin, some extractives, and minerals [28]. Cellulose, hemicellulose, and lignin could create synergistic effects on thermal behavior of the coal [29]. It is believed that H and OH radicals released from biomass during pyrolysis can promote cracking of the aromatic rings of coal [12, 30, 31]. Some researchers also stated that catalytic effects of the minerals in biomass promoted synergy effects between biomass and coal [32]. Yuan et al. [33] conclude that hemicellulose provides strongest promotion effect on the coal conversion during copyrolysis. The holocellulose (cellulose and hemicellulose) components of biomass are mainly responsible for the volatiles of the pyrolysis, which can further produce hydrogen by secondary reactions. Thus, the synergy effect in the presence of legume straw with higher holocellulose and ash content is more significant than that in the presence of pine sawdust with copyrolysis of biomass and coal in a free fall reactor [14]. Lignin in biomass may cause some polymerizing reactions in the low-temperature range, resulting in the formation of resonance stabilized phenoxy radical and other reactive radicals [34–36]. Such radicals are effective and active intermediates to depolymerize coal by cleaving methylene bridges. Nevertheless, the lignin-derived intermediates are short-lived as compared to the time needed for complete coal depolymerization.

Coal is mainly formed as the results of slow metamorphosis of biomass over a long period of time. The degree of that metamorphosis is among the criteria used to determine coal rank. Many researchers [10, 25, 33, 37, 38] found that, during copyrolysis, low rank coals could easily create synergies with biomass and interactions between biomass and low rank coal are more pronounced than those between biomass and high rank coal. They considered that the higher structure similarity between biomass and low-rank coal than that between biomass and high rank coal was the reason. Additionally, as the coal rank decreases, the major pyrolytic decomposition regions of coal shifted to lower temperatures. Biomass reacted in a temperature region close to low-rank coal and this could allow interactions to occur between the components [39]. Simultaneously, hydrogen acceptor ability of low-rank coal was stronger than high-rank coal [40]. During pyrolysis process, the lamellae of the coal cross-linked network is disturbed, causing much fragmentation of coal into hydrogen deficient active sites. Biomass has a higher H/C ratio than coal; the availability of hydrogen around the coal particles would be increased during copyrolysis. There exists hydrogen transfer reaction from biomass to coal. Usually the synergistic positive effect is observed preferentially with low rank coals also due to their stronger ability of capturing hydrogen.

### 2.2. The Influence of Blending Ratio

**Figure 2:**
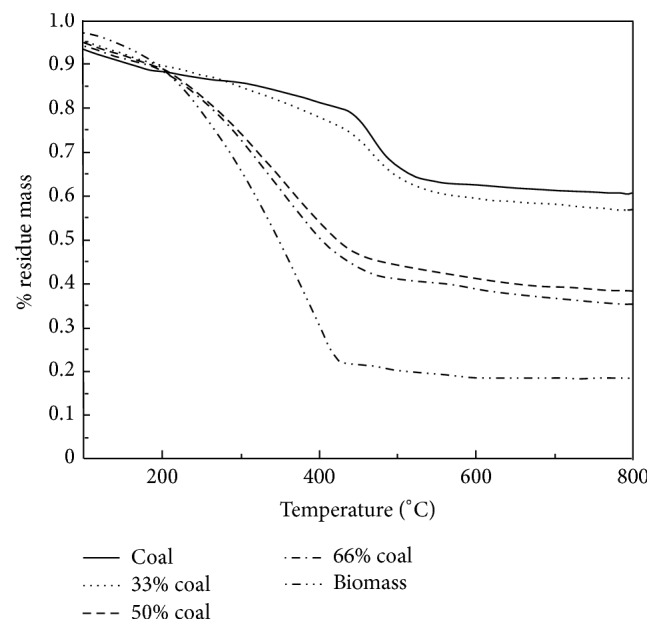
Percent residual mass versus the temperature for raw materials and coal/biomass blends [11].

The proportion of biomass in the blend had a significant influence on product distribution of solid, liquid, and gas [19]. With the increase of biomass blending ratio, the char yield decreases, while the yields of liquid and gas increase [27, 41]. Copyrolysis experiments performed on TG revealed that percent residual mass decreased with increasing biomass content in blends [2, 6, 11, 12, 42, 43]. Typical TG curves for biomass, coal, and their mixtures are presented in Figure 2 [11]. The immobile phase of the coal structure mostly comprises highly cross-linked aromatics, held together by significantly stronger C=C bonds with bond energy of 1000 kJ/mol [19]. These bonds are more difficult to rupture under the heat than the macromolecular structure of cellulose, hemicelluloses, and lignin in biomass, which are linked together by relatively weak ether bonds (R–O–R) with bond energy of about 380–420 kJ/mol. Thus, biomass decomposes much faster than coal. Additionally, biomass undergoes higher weight loss than coal as indicated in Figure 2, and the curve for each biomass/coal blend lies between the curves of the single component.

**Figure 3:**
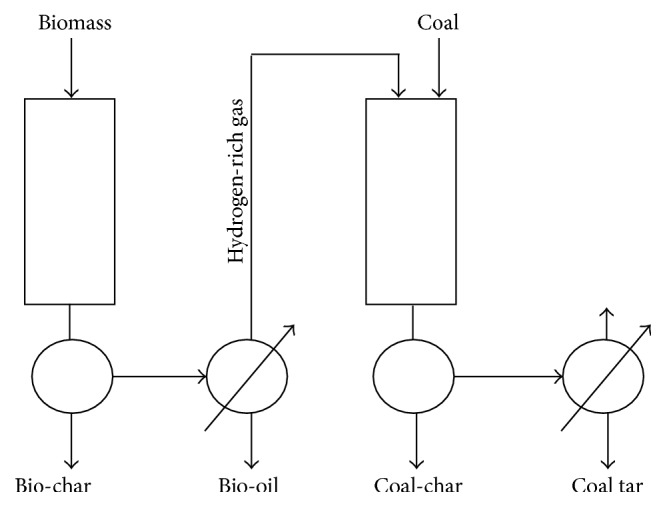
Flow-chart of two-stage copyrolysis process [44].

High thermochemical reactivity and the high volatile content of biomass facilitate the conversion of coal [9, 12]. The degree of synergistic effects was dependent on many factors, that is, coal type, blending ratio, and reactor type [43]. Generally, synergy effect is more pronounced under the higher biomass blending ratio, due to the fact that the presence of sufficient amount of biomass could offer plenty of hydrogen donors to coal [8, 11, 13, 42]. However, degree of synergy effect was not linearly dependent on the amount of biomass in the blends [8, 14, 45]. Since packing density and thermal conductivity of biomass are lower than those of coal, the increase in proportions of biomass would decrease the heating rate of the blends and result in longer releasing time of the volatiles from both biomass and coal [8]. Therefore, OH and H radicals are released more slowly from biomass to enhance the cracking of coal tar [12, 46], and more tar is converted to gaseous products [7]. In addition, biomass char residues formed during copyrolysis are easy to accumulate on the molecules' surface of coal which block the pores of coal molecule through which the volatile matters generated by coal pyrolyzing are driven out. That is to say, the interaction between the solid phases sometimes presents an inhibitive effect on thermal decomposition [6, 11, 47, 48]. In order to avoid accumulating of biomass char on coal surface, Li and Xu [44] proposed two-stage copyrolysis process as illustrated in Figure 3. Fast pyrolysis of biomass and coal was conducted in an individual reactor which was separated from each other in series space, thus making it easy to achieve the best process control of each reactor. The hydrogen-rich gas produced from biomass pyrolysis was then used as a hydrogen source for the coal hydropyrolysis.

### 2.3. The Influence of Heating Rate

The temperature ranges for pyrolysis of biomass and coal differ considerably and it is known that these processes can be distinguished if the heating rate was sufficiently low so only additive behavior can be observed [49]. In view of the different temperature ranges required for the devolatilisation of coal and biomass, the utility of slow heating experiments in looking for synergetic effects appears limited. If the heating rate is increased, the intrinsic devolatilization becomes slower compared with the sample heat up. It could be expected that the pyrolysis processes of biomass and coal could happen simultaneously under very fast heating rate, and, therefore, volatile release from biomass and coal overlapped [13]. Coal pyrolysis yields and products could be different in the case of high heating rates since the reaction atmosphere also involves noninert species [50]. That is to say, copyrolysis of biomass/coal blends at high heating rate favoured the synergism [13, 16, 33, 51]. Suelves et al. [38, 52] report synergy when using pyrolysis-GC (pyroprobe). In this technique, the fuels are blended and contained in a small thin-walled silica tube and a moderate high rate is applied (typical nominal rates of 10^3^ K/s). Yuan et al. [33, 51, 53] conducted rapid pyrolysis of biomass/coal blends on a drop style high-frequency magnetic field based furnace with heating rate higher than 10^3^ K/s. Synergy effect can be found to promote nitrogen release from fuel samples and decrease char-N yields. Zhang et al. [13, 41, 54], who conducted fast pyrolysis experiments in a free fall reactor, also observed appearance of synergy during the copyrolysis of biomass and coal. However, Meesri and Moghtaderi[49] confirmed the lack of synergistic effects on pyrolytic products yields as well as gas composition from pyrolysis of coal/sawdust blends under high heating rate (10^4°^C/s) in a drop-tube reactor, with short residence time and limited particle-particle contact.

When copyrolyzing biomass with coal, coal and biomass are heated together in an inert atmosphere, creating a joint volatile stream and solid char as products. Therefore, the synergistic effect observed during copyrolysis might be due to volatile-volatile interaction and volatile-char interaction [55, 56]. A higher heating rate led to the formation of higher yields of volatile [49, 57, 58]. These increase the probability of gas phase reactions between the volatiles coming from coal and biomass, enhancing the intensity of the synergism. Many authors had confirmed that there was significant synergy in the vapor phase during copyrolysis of coal and biomass [12, 19, 27, 59]. Simultaneously, the volatilization amount of alkali and alkaline earth metallic (AAEM) species at fast heating rates is higher than those at slow heating rate. Such volatilized species may contribute catalytic activity to coal pyrolysis as well as gas phase reactions, resulting in significant synergy or chemical interactions in the vapor phase [9, 12, 60].

### 2.4. The Influence of Temperature

It is known from the literature survey and previous studies that pyrolysis temperature plays an important role on product distribution of coal/biomass blend [8, 11–13, 16]. Pyrolysis of coal yields mainly solid with moderate production of liquid and gas, as opposite to biomass pyrolysis in which liquid and solid equally dominated the products. The experimental yields of solid, liquid, and gas for coal/biomass blend lay between those of coal and biomass; however, appearance of synergy makes them deviate from the calculated yields [12].

With increasing temperature, char yield decreased but volatile matter yield increased [8, 11]. In other words, pyrolysis conversion increased as the temperature increased. Many researchers conclude that biomass may promote devolatilization of the coal at lower temperatures [61]; however, manifestations of the synergies between biomass and coal varied with temperature. Aboyade et al. [5] reported that the interactions occurred between 300 and 500°C, corresponding to the end of biomass devolatilization and the start of coal decomposition. Ulloa et al. [9] considered that interactions detected in the blends were produced at pyrolysis temperatures over 400°C, when most of the components in the blend are devolatilized, and are attributed to secondary reactions that inhibit the formation of char. Park et al. [8] observed that from copyrolysis of sawdust and coal blend in TG the synergy effect to produce more volatiles from coal pyrolysis is pronounced above 400°C. In a fixed bed at isothermal condition, the synergy effects to produce more volatiles appear at 500–700°C, and the maximum synergy exhibits at sawdust blending ratio of 0.6 at 600°C. In a study of Zhang et al. [13], they concluded that the optimum condition for synergy effect in copyrolysis of lignite and legume straw blend in a free fall reactor is 600°C at which enough free radical and hydrogen donors are generated. From the above, it is deduced that 400–600°C may be the optimal temperature range for the synergy occurrence. With further increasing temperature, the synergy effect decreases because of the increased pyrolysis rate and lack of hydrogen donating ability, resulting in the increased retrogressive condensation reactions. Some researchers [8, 12] reported that the increase in temperature to 800°C makes the difference between the experimental yield and the calculated yield decrease or even converge.

### 2.5. The Influence of Reactor Types

Many types of reactors including TG, fixed-bed reactor, fluidized-bed reactor, drop style high-frequency magnetic field based furnace, and free fall reactor have been involved to investigate copyrolysis behavior of biomass and coal. TG is most commonly used. Early reports have concluded that no interactions between the biomass and coal exist during copyrolysis [2–4, 12, 31, 50, 64–66]. Lacks of synergies are mainly due to the low heating rate used in the TG runs (which allowed the different devolatilization phases of both components in the blend to be easily separated) and to the relatively high nitrogen flow rate in the apparatus (which prevented volatile species to remain close to the devolatilizing particles in the crucible, ensuring an inert atmosphere on the sample during the run) [50]. More recent efforts by Aboyade et al. [5], Chen et al. [6], Shui et al. [7], Park et al. [8], Ulloa et al. [9], Yangali et al. [61], and Haykiri-Acma and Yaman [10] have challenged this view, showing that there are indeed significant interactions between coal and biomass fractions during copyrolysis in TG. An overview of the existing literature performed on TG is summarized in Table 1.

Fixed-bed reactors using a relatively large amount of sample would provide intimate contact between neighbouring fuel particles and their volatiles, resulting in the occurrence of the synergy effect for both pyrolysis product yield and gas product compositions [8, 11, 12, 32, 62, 67]. However, intimate contact between biomass and coal particles during copyrolysis does not necessarily mean the occurrence of synergy during copyrolysis [68, 69]. Compared with atmospheric fixed-bed reactor, pressurized pyrolysis and vacuum pyrolysis of coal and biomass also verified the existence of significant synergistic behavior [19, 59]. However, tube furnaces used for pyrolysis usually have long high-temperature zones; volatiles must go through the long high-temperature zone before escaping from the reactors. The extended residence time of intraparticle volatiles allows for increased extraparticle secondary reactions (tar cracking, char-forming) [70]. Therefore, it is difficult to distinguish that the synergies in these reactors are mainly caused by primary pyrolysis process or the second reaction of volatiles [31].

Recently, many varieties of fast pyrolysis reactors have been developed and used to carry out copyrolysis experiment of coal/biomass mixture. These included fluidized beds, drop style high-frequency magnetic field based furnace, and free fall reactor. Some researchers considered that fluidized-bed reactors are not suitable to investigate interactions since near total segregation of sample particles in this apparatus could result in lack of synergies between biomass and coal particles [32]. Yuan et al. [16, 33, 51, 53] conducted rapid pyrolysis of biomass/coal blends on a drop style high-frequency magnetic field based furnace at 600–1200°C, and the nitrogen conversion characteristics of biomass/coal blends were investigated. Synergies can be found to promote nitrogen release from fuel samples and decrease char-N yields under all conditions. Xu et al. [13, 14, 41] had performed copyrolysis of coal and biomass in a free-fall reactor, and they concluded that both the higher blending ratio (around 70 wt.%) and the relatively lower temperature (around 600°C) are more favourable to synergy effects during the copyrolysis of biomass and coal in the free fall reactor. Nowadays, some specially designed reactors, that is, a single-particle reactor system [69], a microfluidized bed reactor [71], and congruent-mass TGA [72, 73], were also applied to study the copyrolysis behaviour of coal/biomass blends.

## 3. Characteristics of Biomass and Coal Copyrolysis Products

The products from copyrolysis of coal and biomass included liquid, char, and gas. The influence of synergy effects on the yield and composition of pyrolysis products would be presented in the following discussion.

### 3.1. Liquid

#### 3.1.1. Liquid Yield

The relatively high content of H_2_ in biomass may play a synergistic role as H_2_ donor during copyrolysis with coal, resulting in more liquid product than that from additive model [11, 13, 14, 56]. Wei et al. [14] reported that the liquid yield was in the range of 25.1–40.9% during copyrolysis of biomass and coal in a free fall reactor from 500 to 700°C, with incremental deviations ranging from 0.9 to 8.0% in the amount of liquid. Onay et al. [11] found that the maximum pyrolysis oil yield reached 39.5% with 5% of lignite mixed with safflower seed during the copyrolysis in a fixed-bed reactor at 550°C; the pyrolysis oil yield increased by about 17% compared to the expected ones. However, in a study by Park et al. [8], even though synergy effect can be found to promote the release of volatiles under all conditions, tar yields are lower than the calculated ones. The lower-than-expected tar yield may be due to the interactions between biomass and coal that promote an additional decomposition of tar to enhance gas yield. As a result, the ratio of tar to the total volatiles decreases.

#### 3.1.2. Liquid Property

Copyrolysis could enhance the transfer of coal hydrogen to valuable petrochemical produced that otherwise would be transferred to molecular hydrogen if coal was pyrolyzed alone [74]. Jones et al. [31] concluded that the pyrolysis oils from biomass/coal blends became poor in aromatics and rich in phenols. Onay et al. [11] reported that the copyrolysis oils contained a greater concentration of single-ring aromatic compounds and aliphatic group rings than biomass pyrolysis oil. Wei et al. [15] also found that the yield of light molecular weight phenols, methylphenol, dimethylphenol, and their derivatives increased at about 5 wt% during the copyrolysis. Song et al. [62] also reported that copyrolysis promoted the yields of phenols and guaiacols in tar. Due to the higher O/C ratio of biomass, the pyrolysis of biomass should produce more oxygenated free radicals. The reactive oxygenated free radicals from biomass react with the unsaturated aromatics from coal and prevent them from recombining to form long chain hydrocarbons [19]. However, high O content of biomass may result in a waste of H, yielding more water. How to transfer H efficiently into oil is a challenge. In order to realize the directional transformation of H and O during copyrolysis of biomass and coal, Zhang et al. [54] constructed a specially designed free fall reactor, where a controlled recontact of volatile with char from primary pyrolysis could be achieved, causing volatile-char interaction, and their results revealed that the recontact of the volatiles-char effectively enhanced tar generation and suppressed water formation during copyrolysis.

### 3.2. Gas

#### 3.2.1. Gas Yield

Sonobe et al. [12] reported that the gas yields are higher than expected from the calculated value based on individual material during copyrolysis of 50 : 50 lignite/corncob blend in a fixed bed reactor, and the gas yield discrepancies took place in the temperature zones of 350–500°C. In the experiments of Yuan et al. [16], who carried out rapid copyrolysis of rice straw and bituminous coal in a high-frequency furnace, they found that gas yield increased with the increasing temperatures. Experimental gas yields were slightly lower than the calculated values in the low temperature range. As the temperature increased, experimental gas yields became higher than the calculated values. They considered that synergy effect promoted gas yields mainly within the high-temperature range. Park et al. [8] found that the gas yield from sawdust and coal pyrolysis at a blending ratio of 0.6 in a fixed bed reactor monotonically increased from 21.2% to 35.7% as the temperature increased from 400 to 800°C, the gas yields are higher than expected by the calculation, and the maximum difference of gas yields between the experimental and calculated ones could reach 6.0% at 400°C. Interactions between biomass and coal promote an additional decomposition of tar to enhance gas yield.

#### 3.2.2. Gas Composition

The abundant gaseous compounds produced from both coal and biomass pyrolysis were mainly CO_2_, CO, CH_4_, and H_2_ [12, 13]. The experimental results showed that the compositions of the gaseous products from blended samples are not all in accordance with those of their parent fuels. Sonobe et al. [12] reported that the experimental yields of CO and CO_2_ of the lignite/corncob blend were more or less identical to the calculated yields at all temperatures. However, Wei et al. [14] found that the experimental yields of CO and CO_2_ are lower than the calculated values. They inferred that, in the copyrolysis, the carbon elements in feedstock have the tendency to move toward tar or char instead of gas. Yuan et al. [16] reported that the experimental CO yields are very close to the calculated values at low temperature. As the temperature increased, experimental yields of CO were higher than the calculated values. Experimental yields of CO_2_ were almost the same as the calculated values. Significant synergy effect in product gas composition was highly pronouncing for CH_4_ formation [12, 14], that is, twice or even three times higher than the calculated values. Sonobe et al. [12] considered that water, one of the major components in biomass volatiles, can be expected to act as a reactive agent to promote the secondary tar cracking producing more CH_4_. It is also suggested that the water could react with the CO to produce active hydrogen, that is, water gas shift reaction (WGSR):

(1) $$\begin{array}{c} CO+H_{2}O={CO}_{2}+H_{2}. \end{array}$$

The new-formed hydrogen produced by WGSR has a higher hydrogenation activity and thus improves the copyrolysis performance [75]. The positive influence of water on copyrolysis of coal and biomass is very useful for practical industrial utilization. It allows the use of the coal and biomass contained large amount of moisture with partial dryness or even without further predrying.

### 3.3. Char

#### 3.3.1. Char Yield

From copyrolysis of biomass and coal, char yields are lower than that from the additive model due to the synergy effect on the additional degradation of the blends by H_2_ supply from biomass pyrolysis with the catalytic effect of inorganic species in biomass ash [8]. There are several factors affecting the extent of the decrease in the char yield. The heat release by secondary reactions is reported to promote the volatilization of primary tars which in turn reduces the char yield [76]. However, some researchers considered that interaction between solid phases presents an inhibitive effect on thermal decomposition during copyrolysis, leading to higher than expected char yield [6, 48]. There are several investigations in which char yields from copyrolysis were found different than expected. Park et al. [8] reported that the maximum difference of char yields between the experimental and calculated ones could reach 8.3% during copyrolysis of sawdust and coal in a fixed bed reactor. Sonobe et al. [12] reported that the solid yield of the lignite/corncob blend was much lower (i.e., 9%) than expected from the calculated value based on individual materials under the range of temperatures studied. In an experiment carried out by Fei et al. [67], the char yield determined experimentally was 1.08–2.88% higher than the calculated values.

#### 3.3.2. Char Property

The pyrolysis conditions determine the chemical composition of the solid products. The volatile-char interaction occurring during copyrolysis has the potential to affect the amount of AAEM in char, the development of char structure, and therefore char reactivity [56]. The biomass blending ratio also significantly affected the copyrolysis char structure evolution, and the addition of biomass could also promote the uniformity degree of the copyrolysis char [43, 63]. AAEM species retained in char during copyrolysis are important catalysts for the gasification/combustion of char. Generally, the in situ pyrolyzed char from the coal/biomass blend exhibited a higher reactivity than that from the coal or the biomass [43, 77, 78]. Zhang et al. [13] found that the CO_2_ reactivity of the chars obtained from the copyrolysis under the higher blending ratio (around 70 wt.%) conditions is about twice as high as those of coal char alone, even higher than those of biomass alone. Nevertheless, Yuan et al. [16] considered that a low biomass/coal mass ratio increases the gasification reactivity of the residual char. The char obtained from copyrolysis can be used in the preparation of active carbon if its pore structure and surface area are appropriate. Additionally, char from copyrolysis of coal and biomass could be used to produce a smokeless solid fuel. Blesa et al. [30] prepared smokeless fuel briquettes from copyrolysis of a low-rank coal and biomass at 600°C with the aim to reduce both the volatile matter and the sulphur content and to increase the high calorific value. Cordero et al. [79] found that the chars resulting from copyrolysis of coal and lignocellulosic wastes show heating values within the range of high-quality solid fuels whereas the ash contents remain in the vicinity of that of the starting coal, which can be used as smokeless.

## 4. Environmental Benefits

Coprocessing of coal and biomass for energy and chemical production will not just reduce fossil-derived CO_2_ emissions, but also limit the discharge of local air pollutants such as SO_*x*_ and NO_*x*_. Blesa et al. [30] studied low-temperature copyrolysis of a low-rank coal and biomass and reported a synergetic effect on the desulphurization of coal. Similarly, Cordero et al. [79] reported that the presence of biomass improved the removal of sulfur from the coal structure when a high sulfur coal was subjected to copyrolysis using waste biomass materials. They explained this mechanism by the hydrogen donor property of biomass, which makes sulfur release from coal easier in the form of H_2_S during copyrolysis. However, some other researchers [10, 46] claimed that additional presence of calcium coming from biomass during copyrolysis should have increased the sulfur fixing potential of char in the form of CaS and CaSO_4_ rather than releasing it and thus leading to a lower sulphur release. The higher the amount of calcium, the higher the amount of sulphur in the cocarbonised material. As for N-containing compounds, Yuan et al. [33] found that, during copyrolysis of biomass and coal, synergies can be found to promote nitrogen release from fuel samples, decreased char-N yields, and increased volatile-N yields.

## 5. Possible Synergistic Mechanisms

Synergistic effects on the copyrolysis can be complicatedly varied depending on the type of blending stock and the pyrolysis condition. Jones et al. [31] outlined some parameters for the synergy. The contact time of fuel particles was well proved to be important for the occurrence of synergy. Some studies also reported the synergistic mechanisms which presumably involved the free radical reactions when lignite and biomass were copyrolyzed. However, knowledge of the synergistic effect remains inadequate. The actual mechanism by which interactions between coal and biomass cause synergy effect during copyrolysis is still not very clear.

### 5.1. Hydrogen Transfer Reaction

One of the main differences in characteristics of biomass as compared to coal is that biomass possesses a higher value of H/C ratio. Under the same pyrolysis condition, the H_2_ yield generated from biomass is about 5–16 times as high as that generated from coal [13]. The pyrolysis of coal may be influenced by the presence of hydrogen-rich light molecules (CO, CO_2_, H_2_, CH_4_, H_2_O, etc.) which are rapidly evolved from biomass at high temperature. These pyrolysis gases may take part in volatile-coal interactions and modify the thermal behavior of coal, especially in the temperature range between 400 and 500°C, where the coal exists in a plastic state.

The transferable hydrogen contained in the coal itself plays an important role for coal plasticity. Actual active methylene carbons such as the naphthenic carbons and ethylene carbons between aromatic moieties might act as hydrogen donor sites. Amount of transferable hydrogen in coal itself was dramatically decreased in the temperature range of 350–500°C [80].

Nevertheless, with copyrolysis of coal with biomass, in the temperature range of 300–600°C, the gas formation rate of H_2_ from biomass pyrolysis was maintained at a constant value [12], thus increasing availability of hydrogen around the coal particles. There are external hydrogen donors to interfere with the chain radical processes between the coal and biomass radicals; chemical interactions therefore occurred. In order to evaluate the degree of hydrogen transfer reaction, two parameters, hydrogen donor ability (HDA) and hydrogen acceptor ability (HAA), of biomass from coal should be evaluated.

### 5.2. Catalytic Effects of AAEM

The presence of AAEM species (mainly K, Na, Ca, and Mg) is in greater abundance in biomass relative to coal. During pyrolysis, volatilization of AAEM species will occur [81]. Such volatilized species may contribute catalytic activity to coal pyrolysis as well as gas phase reactions [9, 12, 60]. To determine the effect of sawdust ash on coal pyrolysis, copyrolysis experiment of sawdust ash/(sawdust ash + coal) blend ratio of 0.2 was carried out on TG by Park et al. [8]. They observed a noticeable DTG peak at around 700°C, which is not observed from the individual thermal decomposition of coal and sawdust. At this temperature, most of volatile matters are removed and residue was mainly composed of coal char and sawdust ash. Therefore, weight loss at this temperature would be the additional decomposition of char by the catalytic effect of inorganic species from sawdust ash. AAEM species present in the biomass, mostly Ca and K, promote demethoxylation reactions. Under normal conditions, these compounds, particularly the methoxyphenols, are precursors to the formation of the aromatic structures of biomass char. However in the presence of aliphatics found in evolved coal volatiles, the methoxyphenols are thought to undergo secondary reactions that produce volatiles instead [5, 9, 45]. At the same time, there have also been suggestions that demineralisation of coal through acid treatment influences the degree of synergy observed [52]. The result may be due to removal of the mineral matter as well as the changes in porosity of coal by the acid treatment. Fei et al. [67] reported that the blends of two original coals synergistically showed a decrease in the tar yield and an increase in the char yield, whereas for the blends using one or two acid-washed coals, the synergy gave rise to increases in both the tar yield and the char yield.

### 5.3. Heat Transfer

Some researchers confirmed that the synergy effect is also caused by heat transfer during the copyrolysis. Thermal decomposition process of lignite was highly endothermic, especially for the reactions occurring between 250 and 475°C during which extensive thermal decomposition of macromolecular chains took place. Thermal decomposition of corncob, on the other hand, was an exothermic process. It is noticed that corncob strongly dominated the behaviour of the blend, observing that the heat profile of the blend followed closely that of corncob especially at temperatures around 250–450°C, suggesting the occurrence of the synergistic activities at around that temperature range [12]. The exothermic heat from corncob pyrolysis could promote the low-temperature thermal decomposition of lignite to form more liquid product.

## 6. Conclusions and Outlook

While facing fossil fuels shortage and severe environmental pollution, biomass, as a clean, storable, and transmittable renewable energy resource, has caught the attention of the world. It is desirable to have coutilization of biomass and coal as a step towards sustainable energy supply system and minimize the impact on the environment by the use of coal. The copyrolysis process and the possible synergy are significantly affected by feedstock type, blending ratio, heating rate, temperature, and reactor types. The synergetic effect could be explained by the transferring of active H radicals from biomass to coal, the catalytic role of AAEM from the biomass, and the heat transfer during copyrolysis. Although the conclusions whether there are synergistic or additive behaviours during copyrolysis are sometimes conflicting, there are some certain laws: higher biomass reactivity and higher structure similarity between biomass and coal could enhance the synergy effect. In addition, under the condition of fast heating rate, the synergistic effects are obvious. Copyrolysis of biomass and coal offers simplicity and effectiveness to produce high-grade pyrolysis oil and higher reactivity char. However, in most published literature, the degree of synergy is judged according to the changes on product yield and product composition. Element migration is the essence of synergy effects during copyrolysis. Therefore, much attention should be paid on the migration regularity and directional control mechanism of hydrogen, oxygen, and other elements during copyrolysis process.

## Figures and Tables

**Table 1:** Co-pyrolysis studies of coal/biomass blends performed on TG.

1st author	HR (°C/min)	Temp. (°C)	Fuels^a,b^	Coal to biomass blend ratio (w/w)	Publishing year
Synergistic behaviors					
Haykiri-Acma [10]	20	900	li-hs	98-96-94-90-80	2007
Chen [6]	10-20-40	1000	semi-cv	30-50-70	2012
Park [8]	15	900	sub-sd	60	2010
Shui [7]	10	800	sub-sd	50	2011
Aboyade [5]	5-10-50	900	hc-bg-cc	90-80-70-60-50	2012
Song [62]	10	1000	sd-li	20-50-80	2014
Ulloa [9]	10-30-50	1200	sub-bit-sd	50	2009
Li [42]	10-15-20-25-30	900	bit-sd	20-40-60-80	2015
Wu [63]	10-20-40	950	ce-bit	25-50-75	2016

Additive behaviors					
Vuthaluru [2]	20	1250	sub-ww-ws	10-20-30-50	2003
Jones [31]	25	900	bit-hvb-li-pw	25-50-75	2005
Sonobe [12]	10	600	li-cc	90-50-10	2008
Kastanaki [4]	10	850	li-oc-fr-cr	95-90-80	2002
Biagini [50]	20	900	hv-lv-sd-ss	15 to 60	2002
Pan [3]	100	900	bl-lq-pc	80-60-40-20	1996
Vamvuka [64]	10	850	li-oc-fr-cr	95-90-80	2003
Sadhukhan [65]	40	1000	li-ww	50-40-10	2008
Idris [66]	10-20-40-60	900	sub-op	80-60-50-40-20	2010

^a^Coals: sub, subbituminous; li, lignite; hvb, high-volatile bituminous; bit, bituminous; semi: semianthracite; bl, black; lq, low-quality; hv, high-volatile; lv, low-volatile; hc, hard coal.

^b^Biomass: ww, wood waste; ws, wheat straw; hs, hazelnut shell; pi, pinewood; cc, corncob; cv: chlorella vulgaris; sd: sawdust; oc, olive cake; fr, forest residue; cr, cotton residue; pc, pine chips; op, oil palm; ss, sewage sludge; ce, cellulose.

## References

[1] Van Krevelen D. W. (1993). *Coal: Typology-Physics-Chemistry-Constitution*.

[2] Vuthaluru H. B. (2004). Thermal behaviour of coal/biomass blends during co-pyrolysis. *Fuel Processing Technology*.

[3] Pan Y. G., Velo E., Puigjaner L. (1996). Pyrolysis of blends of biomass with poor coals. *Fuel*.

[4] Kastanaki E., Vamvuka D., Grammelis P., Kakaras E. (2002). Thermogravimetric studies of the behavior of lignite-biomass blends during devolatilization. *Fuel Processing Technology*.

[5] Aboyade A. O., Görgens J. F., Carrier M., Meyer E. L., Knoetze J. H. (2013). Thermogravimetric study of the pyrolysis characteristics and kinetics of coal blends with corn and sugarcane residues. *Fuel Processing Technology*.

[6] Chen C., Ma X., He Y. (2012). Co-pyrolysis characteristics of microalgae *Chlorella vulgaris* and coal through TGA. *Bioresource Technology*.

[7] Shui H., Shan C., Cai Z. (2011). Co-liquefaction behavior of a sub-bituminous coal and sawdust. *Energy*.

[8] Park D. K., Kim S. D., Lee S. H., Lee J. G. (2010). Co-pyrolysis characteristics of sawdust and coal blend in TGA and a fixed bed reactor. *Bioresource Technology*.

[9] Ulloa C. A., Gordon A. L., García X. A. (2009). Thermogravimetric study of interactions in the pyrolysis of blends of coal with radiata pine sawdust. *Fuel Processing Technology*.

[10] Haykiri-Acma H., Yaman S. (2007). Synergy in devolatilization characteristics of lignite and hazelnut shell during co-pyrolysis. *Fuel*.

[11] Onay Ö., Bayram E., Kocükar Ö. M. (2007). Copyrolysis of seyitömer-lignite and safflower seed: influence of the blending ratio and pyrolysis temperature on product yields and oil characterization. *Energy and Fuels*.

[12] Sonobe T., Worasuwannarak N., Pipatmanomai S. (2008). Synergies in co-pyrolysis of Thai lignite and corncob. *Fuel Processing Technology*.

[13] Zhang L., Xu S., Zhao W., Liu S. (2007). Co-pyrolysis of biomass and coal in a free fall reactor. *Fuel*.

[14] Wei L.-G., Zhang L., Xu S.-P. (2011). Effects of feedstock on co-pyrolysis of biomass and coal in a free-fall reactor. *Journal of Fuel Chemistry and Technology*.

[15] Wei L. G., Zhang L., Xu S. P. (2012). Synergetic effects on tar components from co-pyrolysis of biomass and coal in a free fall reactor. *Journal of Fuel Chemistry and Technology*.

[16] Yuan S., Dai Z.-H., Zhou Z.-J., Chen X.-L., Yu G.-S., Wang F.-C. (2012). Rapid co-pyrolysis of rice straw and a bituminous coal in a high-frequency furnace and gasification of the residual char. *Bioresource Technology*.

[17] Li S., Chen X., Wang L., Liu A., Yu G. (2013). Co-pyrolysis behaviors of saw dust and Shenfu coal in drop tube furnace and fixed bed reactor. *Bioresource Technology*.

[18] Moghtaderi B., Meesri C., Wall T. F. (2004). Pyrolytic characteristics of blended coal and woody biomass. *Fuel*.

[19] Aboyade A. O., Carrier M., Meyer E. L., Knoetze H., Görgens J. F. (2013). Slow and pressurized co-pyrolysis of coal and agricultural residues. *Energy Conversion and Management*.

[20] Collard F.-X., Blin J. (2014). A review on pyrolysis of biomass constituents: mechanisms and composition of the products obtained from the conversion of cellulose, hemicelluloses and lignin. *Renewable and Sustainable Energy Reviews*.

[21] Zhang L., Liu R., Yin R., Mei Y. (2013). Upgrading of bio-oil from biomass fast pyrolysis in China: a review. *Renewable and Sustainable Energy Reviews*.

[22] Lédé J. (2012). Cellulose pyrolysis kinetics: an historical review on the existence and role of intermediate active cellulose. *Journal of Analytical and Applied Pyrolysis*.

[23] Abnisa F., Wan Daud W. M. A. (2014). A review on co-pyrolysis of biomass: an optional technique to obtain a high-grade pyrolysis oil. *Energy Conversion and Management*.

[24] Mushtaq F., Mat R., Ani F. N. (2014). A review on microwave assisted pyrolysis of coal and biomass for fuel production. *Renewable and Sustainable Energy Reviews*.

[25] Haykiri-Acma H., Yaman S. (2010). Interaction between biomass and different rank coals during co-pyrolysis. *Renewable Energy*.

[26] Lee J.-Y., Yoo C., Jun S.-Y., Ahn C.-Y., Oh H.-M. (2010). Comparison of several methods for effective lipid extraction from microalgae. *Bioresource Technology*.

[27] Guo M., Bi J.-C. (2015). Characteristics and application of co-pyrolysis of coal/biomass blends with solid heat carrier. *Fuel Processing Technology*.

[28] Gates B. C., Huber G. W., Marshall C. L., Ross P. N., Siirola J., Wang Y. (2008). Catalysts for emerging energy applications. *MRS Bulletin*.

[29] Wu Z., Wang S., Zhao J., Chen L., Meng H. (2014). Synergistic effect on thermal behavior during co-pyrolysis of lignocellulosic biomass model components blend with bituminous coal. *Bioresource Technology*.

[30] Blesa M. J., Miranda J. L., Moliner R., Izquierdo M. T., Palacios J. M. (2003). Low-temperature co-pyrolysis of a low-rank coal and biomass to prepare smokeless fuel briquettes. *Journal of Analytical and Applied Pyrolysis*.

[31] Jones J. M., Kubacki M., Kubica K., Ross A. B., Williams A. (2005). Devolatilisation characteristics of coal and biomass blends. *Journal of Analytical and Applied Pyrolysis*.

[32] Collot A.-G., Zhuo Y., Dugwell D. R., Kandiyoti R. (1999). Co-pyrolysis and co-gasification of coal and biomass in bench-scale fixed-bed and fluidized bed reactors. *Fuel*.

[33] Yuan S., Chen X.-L., Li W.-F., Liu H.-F., Wang F.-C. (2011). Nitrogen conversion under rapid pyrolysis of two types of aquatic biomass and corresponding blends with coal. *Bioresource Technology*.

[34] Kim J. W., Lalvani S. B., Muchmore C. B., Akash B. A. (1999). Coliquefaction of coal and black liquor to environmentally acceptable liquid fuels. *Energy Sources*.

[35] Coughlin R. W., Davoudzadeh F. (1986). Coliquefaction of lignin and bituminous coal. *Fuel*.

[36] Lalvani S. B., Muchmore C. B., Koropchak J. A., Akash B., Chavez C., Rajagopal P. (1991). Coal liquefaction in lignin-derived liquids under low severity conditions. *Fuel*.

[37] Miao Z., Wu G., Li P., Meng X., Zheng Z. (2012). Investigation into co-pyrolysis characteristics of oil shale and coal. *International Journal of Mining Science and Technology*.

[38] Suelves I., Lázaro M. J., Moliner R. (2002). Synergetic effects in the co-pyrolysis of samca coal and a model aliphatic compound studied by analytical pyrolysis. *Journal of Analytical and Applied Pyrolysis*.

[39] Kastanaki E., Vamvuka D. (2006). A comparative reactivity and kinetic study on the combustion of coal-biomass char blends. *Fuel*.

[40] Kidena K., Murata S., Nomura M. (1996). Studies on the chemical structural change during carbonization process. *Energy and Fuels*.

[41] Quan C., Xu S., An Y., Liu X. (2014). Co-pyrolysis of biomass and coal blend by TG and in a free fall reactor. *Journal of Thermal Analysis and Calorimetry*.

[42] Li S., Chen X., Liu A., Wang L., Yu G. (2015). Co-pyrolysis characteristic of biomass and bituminous coal. *Bioresource Technology*.

[43] Meng H., Wang S., Chen L., Wu Z., Zhao J. (2015). Thermal behavior and the evolution of char structure during co-pyrolysis of platanus wood blends with different rank coals from northern China. *Fuel*.

[44] Li S., Xu S. (2002). Co-pyrolysis of coal and biomass. *Coal Conversion*.

[45] Aboyade A. O., Carrier M., Meyer E. L., Knoetze J. H., Görgens J. F. (2012). Model fitting kinetic analysis and characterisation of the devolatilization of coal blends with corn and sugarcane residues. *Thermochimica Acta*.

[46] Blesa M. J., Fierro V., Miranda J. L., Moliner R., Palacios J. M. (2001). Effect of the pyrolysis process on the physicochemical and mechanical properties of smokeless fuel briquettes. *Fuel Processing Technology*.

[47] Yan W.-P., Chen Y.-Y. (2006). Experimental study on co-pyrolysis characteristics of lignite mixed with biomass mixture. *Journal of Power Engineering*.

[48] Darmstadt H., Garcia-Perez M., Chaala A., Cao N.-Z., Roy C. (2001). Co-pyrolysis under vacuum of sugar cane bagasse and petroleum residue: properties of the char and activated char products. *Carbon*.

[49] Meesri C., Moghtaderi B. (2002). Lack of synergetic effects in the pyrolytic characteristics of woody biomass/coal blends under low and high heating rate regimes. *Biomass and Bioenergy*.

[50] Biagini E., Lippi F., Petarca L., Tognotti L. (2002). Devolatilization rate of biomasses and coal-biomass blends: an experimental investigation. *Fuel*.

[51] Yuan S., Zhou Z.-J., Li J., Chen X.-L., Wang F.-C. (2010). HCN and NH_3_ released from biomass and soybean cake under rapid pyrolysis. *Energy and Fuels*.

[52] Suelves I., Moliner R., Lázaro M. J. (2000). Synergetic effects in the co-pyrolysis of coal and petroleum residues: influences of coal mineral matter and petroleum residue mass ratio. *Journal of Analytical and Applied Pyrolysis*.

[53] Yuan S., Zhou Z.-J., Li J., Chen X.-L., Wang F.-C. (2011). HCN and NH_3_ (NOx precursors) released under rapid pyrolysis of biomass/coal blends. *Journal of Analytical and Applied Pyrolysis*.

[54] Zhang J., Quan C., Qiu Y., Xu S. (2015). Effect of char on co-pyrolysis of biomass and coal in a free fall reactor. *Fuel Processing Technology*.

[55] Weiland N. T., Means N. C., Morreale B. D. (2012). Product distributions from isothermal co-pyrolysis of coal and biomass. *Fuel*.

[56] Wang M., Tian J., Roberts D. G., Chang L., Xie K. (2015). Interactions between corncob and lignite during temperature-programmed co-pyrolysis. *Fuel*.

[57] Mohan D., Pittman C. U., Steele P. H. (2006). Pyrolysis of wood/biomass for bio-oil: a critical review. *Energy & Fuels*.

[58] Demirbas A. (2005). Pyrolysis of ground beech wood in irregular heating rate conditions. *Journal of Analytical and Applied Pyrolysis*.

[59] Yang X., Yuan C., Xu J., Zhang W. (2014). Co-pyrolysis of Chinese lignite and biomass in a vacuum reactor. *Bioresource Technology*.

[60] Zhu W., Song W., Lin W. (2008). Catalytic gasification of char from co-pyrolysis of coal and biomass. *Fuel Processing Technology*.

[61] Yangali P., Celaya A. M., Goldfarb J. L. (2014). Co-pyrolysis reaction rates and activation energies of West Virginia coal and cherry pit blends. *Journal of Analytical and Applied Pyrolysis*.

[62] Song Y., Tahmasebi A., Yu J. (2014). Co-pyrolysis of pine sawdust and lignite in a thermogravimetric analyzer and a fixed-bed reactor. *Bioresource Technology*.

[63] Wu Z., Wang S., Zhao J., Chen L., Meng H. (2016). Thermochemical behavior and char morphology analysis of blended bituminous coal and lignocellulosic biomass model compound co-pyrolysis: effects of cellulose and carboxymethylcellulose sodium. *Fuel*.

[64] Vamvuka D., Kakaras E., Kastanaki E., Grammelis P. (2003). Pyrolysis characteristics and kinetics of biomass residuals mixtures with lignite. *Fuel*.

[65] Sadhukhan A. K., Gupta P., Goyal T., Saha R. K. (2008). Modelling of pyrolysis of coal-biomass blends using thermogravimetric analysis. *Bioresource Technology*.

[66] Idris S. S., Rahman N. A., Ismail K., Alias A. B., Rashid Z. A., Aris M. J. (2010). Investigation on thermochemical behaviour of low rank Malaysian coal, oil palm biomass and their blends during pyrolysis via thermogravimetric analysis (TGA). *Bioresource Technology*.

[67] Fei J., Zhang J., Wang F., Wang J. (2012). Synergistic effects on co-pyrolysis of lignite and high-sulfur swelling coal. *Journal of Analytical and Applied Pyrolysis*.

[68] Montiano M., Díaz-Faes E., Barriocanal C. (2016). Kinetics of co-pyrolysis of sawdust, coal and tar. *Bioresource Technology*.

[69] Wan K., Wang Z., He Y. (2015). Experimental and modeling study of pyrolysis of coal, biomass and blended coal-biomass particles. *Fuel*.

[70] Mok W. S.-L., Antal M. J. (1983). Effects of pressure on biomass pyrolysis. I. Cellulose pyrolysis products. *Thermochimica Acta*.

[71] Mao Y., Dong L., Dong Y. (2015). Fast co-pyrolysis of biomass and lignite in a micro fluidized bed reactor analyzer. *Bioresource Technology*.

[72] Zhang Y., Fan D., Zheng Y. (2016). Comparative study on combined co-pyrolysis/gasification of walnut shell and bituminous coal by conventional and congruent-mass thermogravimetric analysis (TGA) methods. *Bioresource Technology*.

[73] Zhang Y., Zheng Y., Yang M., Song Y. (2016). Effect of fuel origin on synergy during co-gasification of biomass and coal in CO_2_. *Bioresource Technology*.

[74] Lázaro M. J., Moliner R., Suelves I. (1999). Co-pyrolysis of coals and lube oil wastes in a bench-scale unit. *Energy & Fuels*.

[75] Guo Z., Bai Z., Bai J., Wang Z., Li W. (2011). Co-liquefaction of lignite and sawdust under syngas. *Fuel Processing Technology*.

[76] Völker S., Rieckmann T. (2002). Thermokinetic investigation of cellulose pyrolysis—impact of initial and final mass on kinetic results. *Journal of Analytical and Applied Pyrolysis*.

[77] Krerkkaiwan S., Fushimi C., Tsutsumi A., Kuchonthara P. (2013). Synergetic effect during co-pyrolysis/gasification of biomass and sub-bituminous coal. *Fuel Processing Technology*.

[78] Yilgin M., Duranay N. D., Pehlivan D. (2010). Co-pyrolysis of lignite and sugar beet pulp. *Energy Conversion and Management*.

[79] Cordero T., Rodríguez-Mirasol J., Pastrana J., Rodríguez J. J. (2004). Improved solid fuels from co-pyrolysis of a high-sulphur content coal and different lignocellulosic wastes. *Fuel*.

[80] Kidena K., Matsumoto K., Katsuyama M., Murata S., Nomura M. (2004). Development of aromatic ring size in bituminous coals during heat treatment in the plastic temperature range. *Fuel Processing Technology*.

[81] Long J., Song H., Jun X. (2012). Release characteristics of alkali and alkaline earth metallic species during biomass pyrolysis and steam gasification process. *Bioresource Technology*.

